# Previous inguinal hernia surgery does not limit the likelihood of choosing prostatectomy as primary prostate cancer therapy

**DOI:** 10.1038/s41598-024-60451-6

**Published:** 2024-04-30

**Authors:** Mikko Ahtinen, Jaana Vironen, Teemu J. Murtola

**Affiliations:** 1 Department of Surgery, TAYS Cancer Center, Tampere, Finland; 2 Jorvi Hospital, Helsinki University Hospital Abdominal Center, Helsinki, Finland; 3 Faculty of Medicine and Health Technology, Tampere University, Tampere, Finland; 4 Department of Urology, TAYS Cancer Center, Tampere, Finland; 5 Department of Surgery, Tampere University Hospital, Elämänaukio 2, PL 2000, 33521 Tampere, Finland

**Keywords:** Prostate cancer, Inguinal hernia, Prostatectomy, External beam radiation, Urology, Prostate

## Abstract

We evaluated whether previous inguinal hernia repair may affect the choice of prostate carcinoma treatment in a population-based cohort. It has been suggested that previous laparoscopic inguinal hernia repair (LIHR) could limit the subsequent possibility of performing a prostatectomy. Several small studies have suggested otherwise. The study cohort included all new prostate cancer cases in Finland 1998–2015 identified through the Finnish cancer registry. Data on the treatment of prostate cancer and surgical inguinal hernia repairs in 1998–2016 was obtained from the HILMO hospital discharge registry. After linkage, the study cohort included 7206 men. Of these, 5500 had no history of inguinal hernia, 1463 had an open hernia repair, and 193 had a minimally invasive repair (LIHR). Compared to men with no history of hernia repair, those with previous hernia repairs were more likely to undergo prostatectomy over radiation therapy as the primary treatment for prostate cancer HR 1.34 (CI 95% 1.19–1.52). The association did not depend on the method of hernia repair, HR 1.58 (CI 95% 1.15–2.18), in men with previous LIHR. The increased likelihood of choosing prostatectomy over radiation therapy concerns all type prostatectomies. Previous hernia repair is not a limiting factor when choosing treatment for prostate cancer.

## Introduction

Inguinal hernia repair is a common surgical procedure in Western countries. It can be performed with either an open or minimally invasive approach. Synthetic mesh reinforces the inguinal canal structures by local scarring and adhesions in both techniques. In laparoscopic inguinal hernia repair (LIHR), the mesh is placed into the preperitoneal space over the internal inguinal ring and iliac vessels. Mesh can be fixed in place by sutures, glue, or tackers. Alternatively, a self-adhesive mesh can be used. Totally extraperitoneal (TEP) and Transabdominal preperitoneal (TAPP) procedures are called LIHR techniques. LIHR is used primarily in Finland on recurrent or bilateral inguinal hernias^1–3^.

Prostate cancer (Pca) is the most common cancer among men in Europe. Active surveillance (AS) is a widely accepted management strategy for men with low-risk Pca. If AS is selected, it is possible to avoid potential side effects of the Pca treatment without compromising long-term oncological outcomes^4,5^. Radical prostatectomy (RP) and external beam radiation therapy (EBRT) are primary curative-intent treatment methods for localized Pca. Robot-assisted laparoscopic radical prostatectomy (RALP) is the leading operative technique in Finland, as in many other countries worldwide. In addition, pelvic lymph node dissection (PLND) is performed in high- and intermediate-risk cases^6^.

Several case reports and studies were published in the early 2000s describing the difficulties of performing radical prostatectomy and, in some cases aborting the operation in patients with the previous LIHR^7,8^. The reason was severe adhesions obliterating the preperitoneal space. This spurred debate on RP’s safety and feasibility after LIHR. More recent studies have reported that RALP can be performed safely and with good oncological results after LIHR^9–11^. Recently published articles has described feasibility of simultaneous RALP and LIHR operations^12,13^.

Here we explore how previous inguinal hernia repair and its technique are associated with primary PCa treatment at the population level. The primary hypothesis is that patients with a history of LIHR are more often treated with EBRT.

## Material and methods

### Study cohort

The National Research and Development Centre for Welfare and Health administrator permitted to use the national hospital discharge registry HILMO and Finnish Cancer Registry (FCR). The study cohort was formed by linking information from these two registries.

HILMO database registers contain information on diagnoses (as ICD-10 codes) and medical procedures (coded according to Nordic Classification of Surgical Procedures) from all in- and outpatient hospital visits and treatment dates. All Finnish health care units must report information to HILMO. All inguinal hernia repairs among men during 1998–2016 in Finland were identified from HILMO. 1998 was chosen as the ICD-10 coding system was adopted in Finland then. The last year with complete data available was 2016 at the time of data collection. The procedures were identified using Nordic Classification of Surgical Procedures (NOMESCO) codes for the search (Supplementary Table 1). Further inguinal hernia cases were also identified based on ICD-10 codes K40 and K41. We also collected information on common co-morbid conditions: atrial fibrillation (I48), chronic obstructive pulmonary disease (J44), asthma (J45), kidney insufficiency (N18), diabetes (E10-E14), sleep apnea (G47.3), heart condition (I20-I21, I25, I34-I37, I42.0, I42.9, I50), stroke acute or post (I63 and I69), deteriorating brain disease (F00-F03, G30, G20), liver disease (K70-K76) and other cancers besides prostate cancer (Supplementary Table 2). These conditions were chosen as they are included in the Charlson comorbidity index, commonly used to describe the load of comorbidities^14^.

Finnish Cancer Registry was used to obtain information on newly diagnosed prostate cancer cases in Finland between 1998 and 2015, altogether 11,699 cases. Most cancer diagnoses in Finland are included in the FCR database, with 99% coverage^15^. The database contains information on the date of diagnosis, tumor extent at diagnosis, primary treatment, and date of death. Supplementary information on prostate cancer management was added from the HILMO database by searching for codes indicating radiation therapy (KE002, KE009, WF002, WF099), open prostatectomy (KEC00), or laparoscopic prostatectomy (KEC01). Robot-assisted surgery was identified by code ZXC96.

**Figure 1:**
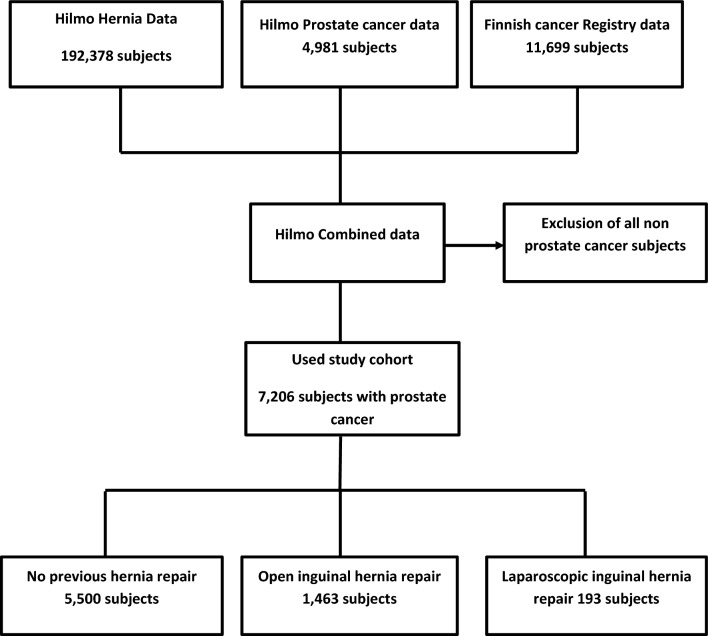
Flowchart of the study. Study population of 7206 Finnish men diagnosed with localized prostate cancer during 1998–2016.

Linking the information from the registries is possible because of the unique personal social security number given to all Finnish residents. After the data linkage study population was limited to men with sufficient data and PCa managed primarily by prostatectomy or radiation therapy, 7206 men. Of these, 5550 had no history of inguinal hernia operation, 1463 had open, and 193 had laparoscopic inguinal hernia operation before PCa management (Fig. 1).

In compliance with Finnish legislation, need for ethics board approval was waived as the study was based entirely on routinely collected registry data^16^. Keeper of each registry that was used as the data source gave approval for the study. The study was carried out by following good clinical practice (GCP) and Declaration of Helsinki.

### Data analysis

Statistical Package for Social Science for Windows, version 26.0 (SPSS), was used for all statistical analyses. P-value < 0.05 was set for statistical significance.

### Ethical approval

For this type of article, informed consent is not required. When The National Research and Development Centre for Welfare and Health permitted to use the registers, they have inspected the research plan. During that process, they checked the plan to fulfill the research's ethical standards as well as the requirements of the Finnish legislation. The need for informed consent was waived as the study used routinely collected register data, which is standard practice in Finland. Tampere University Hospital research unit and the local ethics committee were informed of the permission to use the registers .The ethics committee waived their process and did not demand any actions to be taken.

## Results

### Population characteristics

Radical prostatectomy was equally common in men with inguinal hernia repair and those without it (69% vs. 62%, respectively). Age at diagnosis was similar among men with or without hernia repair. Comorbidities were divided equally. Subjects with a history of inguinal hernia repair often had other cancers besides prostate cancer, atrial fibrillation, diabetes, and heart disease. The open hernia group had less often localized cancer, but the number of cases with missing information was higher (Table 1).

**Table 1:** Population characteristics.

	Primary inguinal hernia surgical treatment		
	None	Open	Laparoscopic
No. patients	5550	1463	193
Prostatectomy	3443 (62.0%)	1011 (69.1%)	140 (63.8%)
ERBT	2107 (38.0%)	452 (30.9%)	53 (36.2%)
Age at diagnosis; median (IQR)	67 (62–72)	68 (63–74)	68 (63–74)
Atrial fibrillation no	64 (1.2%)	51 (3.5%)	5 (2.6%)
Asthma no	37 (0.7%)	21(1.4%)	2 (1.0%)
COPD no	16 (0.3%)	10 (0.7%)	1 (0.5%)
Kidney disease no	7 (0.1%)	1 (0.1%)	1 (0.5%)
Diabetes no	68 (1.2%)	34 (2.3%)	7 (3.6%)
Sleep apnea no	10 (0.2%)	12 (0.8%)	1 (0.5%)
Heart disease no	96 (1.7%)	54 (3.7%)	6 (3.1%)
Stroke no	17 (0.3%)	6 (0.4%)	1 (0.5%)
Deteriorating brain disease no	15 (0.3%)	11 (0.7%)	1 (0.5%)
Liver insufficiency no	2 (0.0%)	2 (0.1%)	0 (0.0%)
Other cancer than prostate cancer no	12 (0.2%)	24 (1.6%)	3 (1.6%)
Cancer extent no			
Localized	3681 (67.3%)	690 (52.9%)	95 (64.3%)
Locally advanced	324 (5.9%)	58 (4.4%)	9 (5.6%)
Metastatic	608 (11.1%)	217 (16.6%)	33 (12.3%)
Unknown or information missing	856 (15.7%)	339 (26.0%)	38 (17.7%)

The study population is 7206 Finnish men diagnosed with prostate cancer from 1998 to 2016.

### Likelihood of radicalprostatectomy as a primary prostate cancer treatment by previous inguinal hernia repair history

Subjects with a history of inguinal hernia repair were more likely to be treated with RP rather than EBRT as the primary treatment modality HR 1.34 (CI 95% 1.19–1.52). The risk association was strongest for robot-assisted laparoscopic prostatectomy when previous hernia repair had been done using the LIHR technique; HR 9.10 (CI 95% 6.20–13.53). However, the size of this subgroup was small. RALP was more popular than EBRT after inguinal hernia repair with both open and LIHR techniques (Table 2).

**Table 2:** Effect of inguinal hernia repair on choosing prostate carcinoma treatment.

Likelihood of undergoing external beam radiation therapy instead of radical prostatectomy as prostate cancer management						
Prostatectomy method	Prostatectomy, any method		Open prostatectomy		Robot-assisted prostatectomy	
Hernia repair method	N EBRT/prostatectomy	HR (95% CI)	N EBRT/prostatectomy	HR (95% CI)	N EBRT/prostatectomy	HR (95% CI)
No hernia repair	2107/3443	Ref	2107/993	Ref	2107/294	Ref
Open hernia repair	452/1011	1.34 (1.19–1.52)	452/709	3.24 (2.81–3.73)	452/386	5.92 (4.92–7.13)
Laparoscopic hernia repair	53/140	1.58 (1.15–2.18)	53/94	3.69 (2.61–5.22)	53/69	9.10 (6.20–13.53)

Hazard ratios were adjusted in a multivariate model for age and comorbidities (atrial fibrillation, asthma, COPD, kidney disease, sleep apnea, heart disease, stroke, deteriorating brain disease, liver insufficiency, and other cancer than prostate cancer).

*HR* hazard ratio, *CI* confidence interval.

### Subgroup analyses

We estimated whether the method or number of previous hernia repairs would modify the likelihood of choosing radiation therapy over prostatectomy. The likelihood of prostatectomy was similar among men with a history of laparoscopic bilateral hernia repair HR 1.93 (CI 95% 1.09–3.45) and men with open bilateral inguinal hernia repair HR 2.39 (CI 95% 1.38–4.10). The number of subjects with a history of bilateral hernia was low in both open (21 EBRT and 63 RP) and laparoscopic (19 EBRT and 47 RP) repair groups (Table 3).

**Table 3:** Association between previous bilateral inguinal hernia repairs (**A**) and between multiple inguinal hernia repairs (**B**) by a primary treatment method in localized prostate cancer.

Likelihood of undergoing external beam radiation therapy instead of radical prostatectomy as prostate cancer management		
Status of hernia repair	Prostatectomy, any method, N EBRT/prostatectomy	HR (95% CI)
(A)		
No hernia repair	170/222	Ref
Open bilateral hernia repair	21/63	2.39 (1.38–4.10)
Laparoscopic bilateral hernia repair	19/47	1.93 (1.09–3.45)
(B)		
No hernia repairs	2107/3443	Ref
One previous hernia repair	435/1010	1.40 (1.23–1.58)
Two or more previous hernia repairs	70/141	1.20 (0.90–1.62

Hazard ratios were adjusted in a multivariate model for age and comorbidities (atrial fibrillation, asthma, COPD, kidney disease, sleep apnea, heart disease, stroke, deteriorating brain disease, liver insufficiency, and other cancer than prostate cancer).

*HR* hazard ratio, *CI* confidence interval.

Among participants, who had two or multiple inguinal hernia repairs before PCa treatment, it was still more likely to have prostatectomy over EBRT HR 1.20 (CI 95% 0.90–1.62). Again, the number of subjects in this subgroup was low, with 70 cases in EBRT and 141 cases in prostatectomy groups (Table 3).

Further, we studied whether the increase in minimal-invasive techniques in inguinal hernia repair during our study period may modify the likelihood of choosing RP. Since LIHR has become more popular since 2000, the study population was stratified by PCa diagnosis date as early (1.1.1998–31.12.2006) and late (1.1.2007–31.12.2015) cases. Timing of PCa diagnosis did not significantly modify the choice of prostate carcinoma treatment among subjects with previous open hernia repair; HR 1.65 (CI 95% 1.33–2.05) in the early group versus HR 1.38 (CI 95% 1.16–1.62) in the late group. The outcome was similar among subjects with a history of laparoscopic hernia repair, although group size was small in both early and late groups (Table 4).

**Table 4:** Likelihood of choosing radiation therapy instead of prostatectomy as a primary prostate cancer treatment in men with a history of previous hernia repair.

Likelihood of undergoing external beam radiation therapy instead of radical prostatectomy as prostate cancer management				
PCA dg date	Early 1.1.1998–31.12.2006		Late 1.1.2007–31.12.2015	
Hernia repair method	N EBRT/prostatectomy	HR (95% CI)	N EBRT/prostatectomy	HR (95% CI)
No hernia repair	1060/1789	Ref	1019/1601	Ref
Open hernia repair	128/366	1.65 (1.33–2.05)	252/558	1.38 (1.16–1.63)
Laparoscopic hernia repair	11/37	1.96 (0.99–3.87)	36/91	1.55 (1.05–2.31)

Analysis stratified by timing of prostate cancer diagnosis to evaluate the effect of evolving surgical technique. Hazard ratios were adjusted in a multivariate model for age and comorbidities (atrial fibrillation, asthma, COPD, kidney disease, sleep apnea, heart disease, stroke, deteriorating brain disease, liver insufficiency, and other cancer than prostate cancer).

*HR* hazard ratio, *CI* confidence interval.

## Discussion

Our population-based cohort study results do not support the assumption that previous hernia repair would limit surgical management of PCa. Even LIHR is not a limiting factor when choosing a treatment modality for Pca patients. Conversely, men with a history of hernia repair were more likely to be managed with prostatectomy than EBRT. It may reflect this group’s general fitness for surgical operations and positive attitudes towards procedures.

In the early 2000s, patients were warned that the LIHR technique would limit the possibility of performing a prostatectomy later. Although this may be true for open prostatectomy, most recent studies have shown that RALP could be safely performed with good oncological results after previous inguinal hernia repai^9–11^. Mini-invasive, especially robot-assisted laparoscopic prostatectomy, has become the preferred choice over the open technique. During the same period, the number of LIHR operations has risen constantly. Our subgroup analyses suggest that this trend may not affect the likelihood of undergoing prostatectomy to any significant degree. LIHR has had a very modest effect on RALP when considering operative, hospital stay, blood loss, or complications^17–19^. However, PLND is more demanding and, in some cases, impossible to perform^20^. Nevertheless, it does not limit choosing radical prostatectomy as the primary management. Recently studies have been published about feasibility of simultaneous RALP and LIHR. Naturally this would not aid those subjects that have had the LIHR operation done previously^12,13^.

Register-based studies are a good imprint of real life. Our study population is based on the entire population of Finland. Thus generalizability of our results in the general Nordic population is good. We confirmed previous results that inguinal hernia repair before prostate carcinoma does not limit later surgical treatment options.

Our study has several limitations. We did not have data on whether lymphadenectomy was performed during prostatectomy, even though we had information on cancer extent. The registries we used did not contain information on BMI. A lengthy study period means there has been a transition in surgical techniques. Furthermore, some participants may have had an inguinal hernia repair before 1998. It may have caused bias towards the null but does not limit our inference on the increased likelihood of choosing prostatectomy.

## Conclusions

Our population-based cohort study confirms that open or laparoscopic inguinal hernia operation does not limit prostatectomy use for PCa management. In clinical practice it would mean that Pca treatment can be tailored according to the patient, comorbidities and cancer.

### Supplementary Information

Supplementary Tables.

## Data Availability

The dataset of this study is not publicly available. However, on reasonable request, derived data supporting the findings of this study are available from the corresponding author after approval from Finnish Social and Health Data Permit Authority Findata.
