# Supplementary material for: Previous inguinal hernia surgery does not limit the likelihood of choosing prostatectomy as primary prostate cancer therapy
Source: Sci Rep. 2024 Apr 30;14:9943. doi: 10.1038/s41598-024-60451-6 (PMC11061137; doi:10.1038/s41598-024-60451-6)
Supplement: Supplementary file 1 — Supplementary Tables. [file 41598_2024_60451_MOESM1_ESM.docx]

**Supplementary table 1.** The procedures of inguinal and femoral hernia operations that were identified from registers by using Nordic Classification of Surgical Procedures (NOMESCO) codes.

| **Hernia /operation** | **open** | **minimal-invasive** |
| --- | --- | --- |
| **Inguinal** | JAB10, JAB20, JAB30, JAB40, JAB90, JAB92, JAB93, JAB94, JAB95 JAB96 | JAB11, JAB12, JAB91, JAB97 |
| **Femoral** | JAC10, JAC30, JAC40, JAC90, JAC92, JAC93, JAC94, JAC 95, JAC96 | JAC11, JAC12, JAC91, JAC97 |

**Supplementary table 2.** The other cancers beside prostate cancer according to ICD-10 codes

| **other cancers beside prostate cancer (ICD-10 codes)** |
| --- |
| C15-C16, C18-C25, C34, C39, C60, C62, C64-C67, C73-C74, C81-C83, C85, C88, C90-C93 and C95 |

Table legend, for both tables :

**Supplementary tables expressing used NOMESCO and ICD-10 codes**
